# Women’s narratives of experiences, drivers and consequences of mistreatment during maternity care in western Ethiopia

**DOI:** 10.1371/journal.pone.0313217

**Published:** 2024-12-13

**Authors:** Habtamu Kasaye, Vanessa Scarf, Annabel Sheehy, Kathleen Baird

**Affiliations:** 1 Collective for Midwifery, Child and Family Health, School of Nursing and Midwifery, Faculty of Health, University of Technology Sydney, Ultimo, NSW, Australia; 2 Department of Midwifery, School of Nursing and Midwifery, Institute of Health Sciences, Wollega University, Nekemte, Oromia, Ethiopia; McGill University, CANADA

## Abstract

**Background:**

The mistreatment of women during maternity care hinders quality care globally and deter women from seeking health services. To implement necessary actions, it is essential to explore instances of mistreatment, their factors and negative outcomes. This study explores the narratives of mistreatment experienced by women, its drivers, as well as the consequences of mistreatment.

**Methods:**

We conducted a descriptive qualitative study among women who had received maternity care at East Wollega Zone, Ethiopia. Data were obtained through in-depth interviews with purposively selected participants in Afan Oromo, each lasting, on average, 30 to 60 minutes. Interviews were conducted within three months of childbirth and discontinued upon reaching data saturation at seventeen interviews. All interviews were audio recorded, transcribed, translated into English, coded using NVivo 12 and analysed through thematic and framework analysis.

**Result:**

Three main themes were identified in this study: experiences, drivers, and consequences of mistreatment of women during maternity care. The narratives of mistreatment fell into two sub-themes: interpersonal abuse and mistreatment in the process of care. Women described experiencing physical and verbal abuse, stigma, and discrimination, as well as neglect and abandonment, violations of privacy and confidentiality, and health facility failures related to resource limitations. These forms of mistreatment were perceived to arise from a complex interaction of factors at an individual, interpersonal, and facility level, as well as broader health system and societal norms, such as gender inequality. The identified consequences of mistreatment included fear of future childbirth, negative perceptions towards health facilities and healthcare providers, switching to home birth, and psychological stress.

**Conclusions:**

This qualitative study presents women’s first-hand experiences of mistreatment in health facilities, highlighting various forms stemming from interpersonal interactions and systemic deficiencies in care quality. These experiences lead to significant negative consequences and implications on service delivery. The findings underscore the importance of understanding the complex factors driving mistreatment, extending beyond individual healthcare providers’ behaviours to macro-level health system issues and general violence against women in society. This emphasises the importance of applying a systems-thinking approach to address the abuse and suffering women experience during maternity care in health facilities.

## Introduction

In recent decades, a significant shift has transpired in the discourse regarding the mistreatment of women and the quality of maternal health services instigated by pivotal research and international initiatives. Bowser and Hill’s [1] global investigation, the establishment of the Respectful Maternity Care (RMC) Charter by the White Ribbon Alliance [2], and the World Health Organization’s (WHO) condemnation of disrespect and abuse [3], all signal a marked focus on systemic drivers of mistreatment of women experienced in childbearing and integration of RMC in WHO guidelines for positive childbirth experiences [4]. Despite these activisms against the mistreatment of women during maternity care in health facilities, it remains a pervasive global issue for women [5].

Any childbearing woman can experience mistreatment when using health services [3], and has been reported to affect up to 44% of women in Sub-Saharan Africa [6], two out of every five women in Latin America [7], every five women in Europe [8], every six women in the United States of America [9], and every ten women in Australia [10]. The widespread mistreatment of women highlights the urgent need for targeted interventions at individual and systemic levels. Focusing exclusively on system-level improvements risks overlooking the essential role of personal accountability in ensuring respectful care [11]. Addressing and eliminating the mistreatment of women in maternity services requires a multifaceted approach involving systemic changes, policy interventions, and a cultural shift [12]. Indeed, this underscores the requirement for individual health services to examine the unique contextual factors influencing instances of mistreatment within their respective settings. This paper focuses on the issue of mistreatment of women during maternity care, with a specific emphasis on its occurrence and consequences in Ethiopia.

In Ethiopia, the prevalent mistreatment of women during maternity care, which ranges from 2.0% to 79.0% [13], undermines the national commitment to reducing maternal mortality [14] by impeding the quality of essential, safe and respectful maternity care and potentially deterring women from seeking health services [15, 16]. Prior research in this domain has primarily concentrated on gaining insight through quantifying women’s subjective experiences [17]. To understand its nature, origins, and consequences comprehensively, it is crucial to consider how women describe their experiences throughout the entire continuum of maternity care rather than focusing solely on childbirth. Despite the essentiality and significance of women’s perspectives, there needs to be an adequate exploration of the drivers of mistreatment beyond the level of the individual and the consequences of the mistreatment of women.

Given the nuanced and multifaceted nature of mistreatment, comprehensive research must now encompass all relevant stakeholders, including women, healthcare providers, senior management of healthcare facilities and the community at large [18]. To present this comprehensive perspective in a manner that centres women’s experience without attributing blame to any specific group [19], we adopted the term ‘mistreatment of women’ as well as in other publications that explore different facets of the mistreatment of women [15, 20]. Employing a qualitative design, this paper presents the findings of women’s expectations of healthcare facilities, experiences of mistreatment, the underlying drivers, and perceived consequences. These insights were analysed using thematic analysis and deductively by integrating the Socioecological Framework for violence against women [21] and WHO quality of care frameworks [22].

## Methods

### Study context, design and frameworks

This paper presents the qualitative segment of a broader research inquiry that investigated the mistreatment of childbearing women in Ethiopia. As part of a nested convergent mixed-method approach, a qualitative study was conducted in East Wollega Zone, Oromia Regional State, Western Ethiopia, from September 2022 to December 2022. This region in Ethiopia is characterised by diverse Agro-ecological zones and geographically challenging topography, impacting the availability and access to health infrastructure, particularly for rural people, where nearly 83% of the population is projected to live in rural areas in 2022 [23], with an estimated 1.80 million people in East Wollega Zone in 2022, representing 4.5% of the regional and 1.7% of the national population [23, 24].

Oromia region has shown subpar performance in maternal and child health, as evidenced by statistics indicating that only 71% of pregnant women receiving antenatal, 44% of births were attended by skilled attendants, which mainly comprised of midwives both at health centres and hospitals, and 56% of women giving birth without the assistance of midwives or other trained birth attendants. Although zonal-level data was not publicly available, regional data shows that the area has the third-highest total fertility rate in the country, with 5.4 births per woman and an average household size of 5.2 people. In 2022, an estimated 3.9 million live births occur in the country, with approximately 54,000 occurring in the East Wollega Zone [25, 26]. Of these, around 23,760 births took place in health facilities, with an annual average of 3,264 at Nekemte Specialized Hospital and 3,072 at Wollega University Referral Hospital, while the rest occurred at three district hospitals and 68 health centres [27]. In our study, the majority of births (78.4%) occurred in a hospital, with 15.3% taking place in health centres and 1.7% in private clinics [15].

Mistreatment during childbirth is conceptualised in this study as it parallels the violence against women in the community, emphasising the seriousness of the issue, particularly within the context of a critical period in a woman’s life—pregnancy and birth [11]. This framing positions mistreatment as actions or behaviours that inflict physical, emotional, or psychological harm and violations of the dignity and rights of women [3]. Woman-centred principles underpin the examination of the mistreatment of women and mandate that maternity care provision is both respectful and safe [4]. To explore how mistreatment arises from both abusive actions by healthcare providers and systemic failures in maternity care, we drew upon two conceptual frameworks: The Integrated Socioecological Framework for Violence Against Women and the WHO Quality of Care Framework [22]. Both tools were used to understand the complex interplay of individual, interpersonal, community, and societal factors of the mistreatment of women in health facilities, and the rationale for selecting these frameworks are discussed in further detail below.

### Socioecological framework of violence against women

Mistreatment of women within the context of childbearing is challenging to investigate because of the numerous external factors beyond an individual woman’s immediate context that indirectly influence an individual’s experiences of pregnancy, birth and early mothering [18]. Investigating a complex phenomenon using a concept that involves an interplay of various levels allows for a comprehensive understanding of the multifaceted factors contributing to the phenomenon and recognises the interconnectedness and mutual influence among the different levels [28].

**Fig 1:**
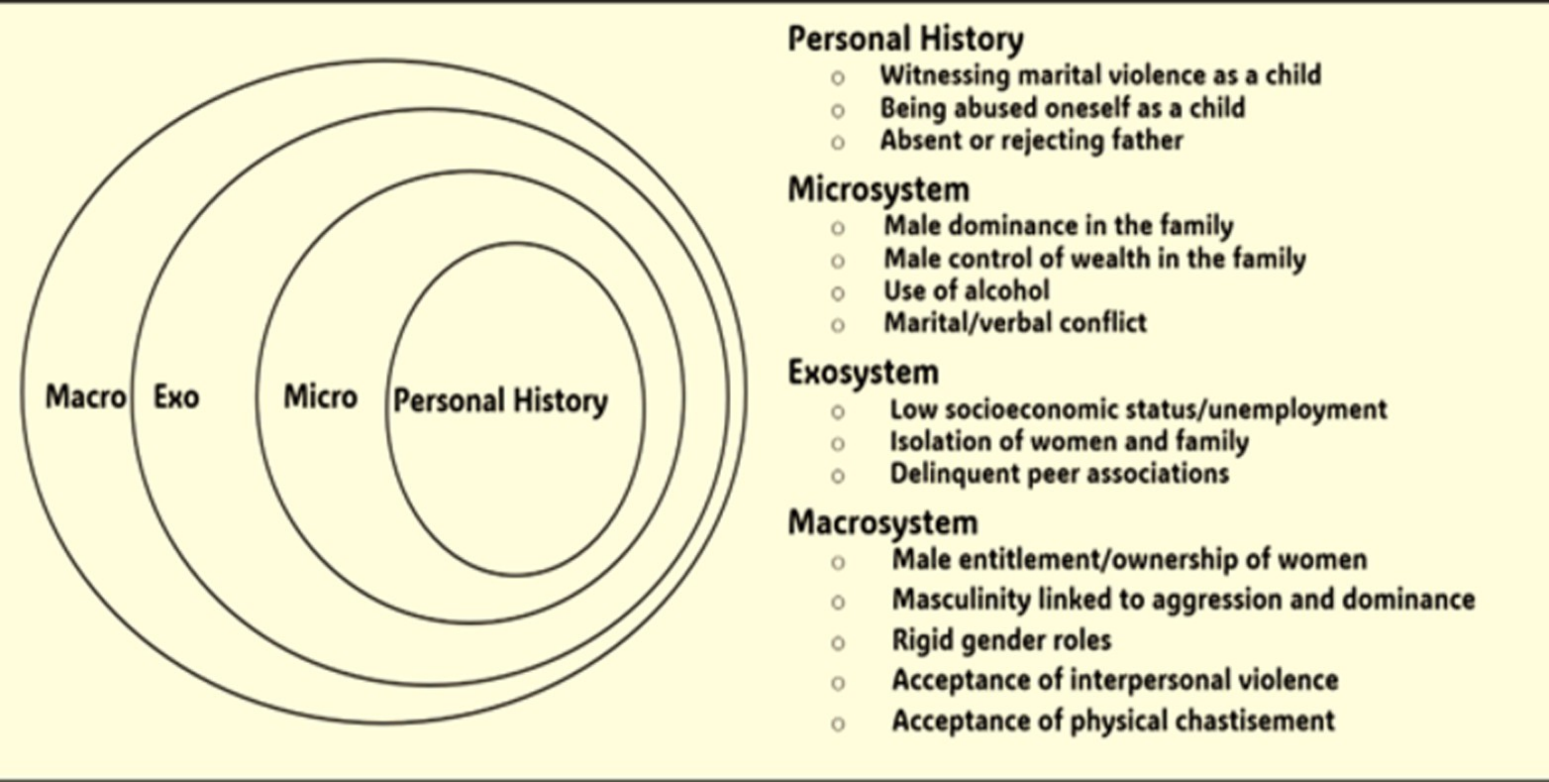
Socioecological framework for the violence against women.

The Socioecological Framework of Violence Against Women conceptualises violence against women as an interplay of various levels of influence, these being personal history, microsystem/interpersonal, mesosystem (health facilities), exo-system/health system, and macrosystem/societal domains [21]. Fig 1 presents a pictorial representation of the framework. Adopting the Socioecological Framework enables a holistic perspective that recognises the multifaceted nature of mistreatment across health system, community, health facility, interpersonal, and individual levels. This framework facilitates the identification of drivers of mistreatment, contributing to the development of multifaceted interventions at various levels.

### WHO’s Quality of Care Framework

**Fig 2:**
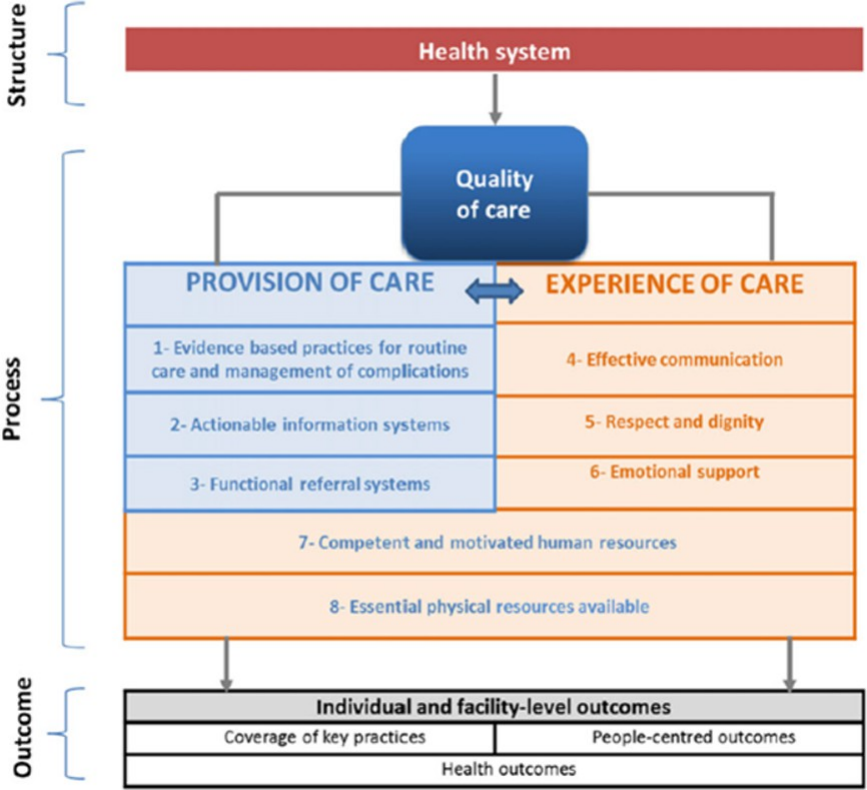
WHO Quality of Care Framework for maternal and newborn health.

The WHO Quality of Care Framework conceptualises that the quality of healthcare for women and their babies emanates from structural and content conditions of the health system [22]. This framework (see Fig 2) allows for evaluating the quality of women’s and newborn healthcare services by emphasising safe and evidence-based woman-centred practices. Its relevance to mistreatment lies in its direct linkage of a woman’s care experience to the care provided, positioning mistreatment as a potential outcome of engaging with health services [22].

**Fig 3:**
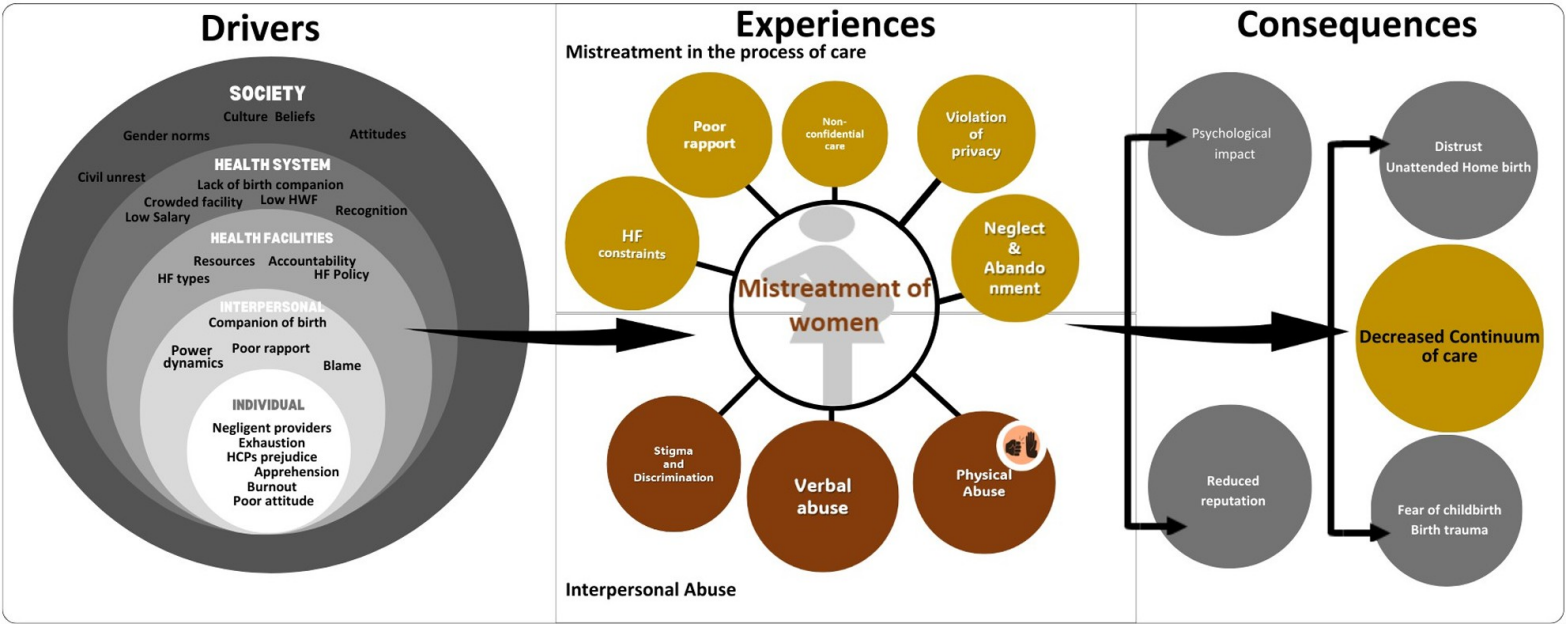
Framework illustrating the mistreatment of women during maternity care, synthesised by integrating components from the Socioecological and WHO Quality of Care Frameworks.

The Socioecological Framework provides a broader societal context for understanding violence, presenting how social structures, institutions, and interactions contribute to violent behaviour. In contrast, yet complementing the Socioecological Framework, the WHO Quality of Care Framework focuses more specifically on the organisational and structural aspects of healthcare, such as the process of care and desired health outcomes, ensuring a focus on evidence-based practices, safety, woman-centred care, timely access, and equitable delivery. The framework we developed by integrating these two frameworks is visually presented in the results section (Fig 3), with the corresponding findings detailed in each part.

### Recruitment and sampling

The qualitative data collection involved purposively selected participants from the quantitative phase of the larger study, conducted within three months of birth [15]. The participants had received at least one component of the continuum of care—antenatal, birth, or postnatal care. Healthcare providers were also interviewed; however, their results have been compiled in a separate report and are included in this manuscript. Participants included women from diverse backgrounds (rural and urban settings, younger and older, multiparous and primiparous) who were recruited and interviewed to provide a thorough understanding of the phenomenon. Telephone interviews were conducted at mutually agreeable times and were chosen due to the large geographical area and the ongoing civil conflict in Ethiopia. Data collection ceased after seventeen interviews when data saturation was reached, as no new insights emerged from adding further participants.

### Data collection instruments

A semi-structured interview guide, aligned with the research aims, was formulated to elicit detailed insights from participants concerning the mistreatment of women, its drivers, and the consequences of mistreatment. The interview guide for both the women and healthcare professionals comprised four domains focusing on (1) expectations from healthcare facilities during maternity care; (2) experiences and perceptions of mistreatment from healthcare providers in recent maternity care; (3) perceived factors influencing mistreatment; and (4) potential impacts of mistreatment on service utilisation.

### Data collection procedures

Interview participants were informed of the voluntary nature of their participation, provided with a participant information sheet, and requested to complete consent forms if they wished to participate. Assurances of confidentiality were given, emphasising their ability to terminate the interview at any point without repercussions. Participants were also informed that the study’s researchers were independent of healthcare facilities, and their participation would not impact their healthcare utilisation. HK conducted in-depth interviews with women; all interviews were conducted in the Afan Oromo language. HK speaks the same language as participants, is part of their ethnic group, and deeply understands the local service delivery system and the sociocultural context. HK was a PhD student at the time of data collection, trained in qualitative research under the supervision of experienced qualitative researchers (KB, VS and AS). Following data collection, the audio-recorded data were transcribed and translated into English to analyse and report the findings in collaboration with English-speaking authors (KB, VS, and AS). On average, each interview lasted between 30 and 60 minutes.

### Analysis

This study was analysed thematically using the Braun and Clarke data analysis framework [29]. Thematic analysis is an inherently flexible method and helps identify key themes, richly describing large bodies of qualitative data and highlighting similarities and differences in experience [29, 30]. The themes were then guided by the frameworks mentioned above.

The interviews were transcribed verbatim into Afan Oromo and then translated into English for further analysis. The transcripts were read in their entirety whilst listening to the audio recordings to check for accuracy and begin comprehending and understanding this large data set. Line-by-line coding of the transcripts was conducted on sub-samples exported to the qualitative data management program NVivo (Version 12.) for coding. Descriptive coding, in-vivo and process coding of the participants’ data were performed to the development of initial coding as recommended by Saldaña [31]. The inductive themes identified from the data and deductive analytic approaches from the interview guide were employed. Hence, the parts related to the mistreatment experiences employed descriptive analysis, while the Socioecological Framework for Violence Against Women [21] was used to explore the perceived drivers of mistreatment in health facilities.

### Reflexivity

The primary researcher (HK) is a midwifery lecturer at the public university within the study area, with over a decade of midwifery experience. The supervisors are also academic supervisors in midwifery but are outsiders to the study context, having no prior exposure to the study area or its participants. The primary author conducted this research as part of his PhD studies in Midwifery. None of the participants had a prior relationship with the interviewer, the primary author. Participants were made aware of the purpose of the research and that their responses would be kept confidential before the interviews commenced.

The study took place during a period of civil unrest in the area, significantly impacting the conditions for research. The security situation posed serious challenges, making it difficult to move freely between districts and execute the study as initially planned, especially in engaging participants from rural areas. This contributed to the decision to perform the interviews over the phone for safety reasons, and this methodological adaptation raises awareness of potential limitations, such as the reduced capacity to capture non-verbal cues. This shift prompts reflection on the balance between accessibility and nuanced data collection in challenging contexts.

While conducting this study, even though the researcher perceives the presence of contributing health system and individual factors in the area as an insider, the primary author and the rest of the research team firmly believe there should be no justification for instances of mistreatment. It is not something we should tolerate; instead, it requires collaborative efforts from all local and international actors.

### Ethics approval and consent to participants

All participants were provided with information regarding the study in their preferred language to enable understanding. Participants were given sufficient time to ask questions and reflect on the information provided. Written informed consent was received from each participant, and they were informed that they were free to discontinue or withdraw from the study without any negative consequences. Ethical approval was granted from the local Institutional Review Committee in Ethiopia, Wollega University, and the Human Research Ethics Committee at the University of Technology Sydney, with the approval number of UTS HREC REF NO. ETH21-6587.

This study was reported in accordance with the consolidated criteria for reporting qualitative research (COREQ) [32].

## Results

### Overview

Overall, seventeen interviews were conducted with women. The women’s ages ranged from 20 to 33 years, including primiparous and multiparous women (2 to 4 births) and were from three districts (Nekemte, Diga and Wayyu Tuka) (Table 1).

**Table 1:** Demographic characteristics of the women who participated in the in-depth interview (n = 17).

	Categories	Frequency (%)
Age	18–24	4(23.5)
	25–29	7(41.2)
	30 and older	6(35.3)
Educational status	Primary	4(23.5)
	Secondary	6(35.3)
	College and higher	7(41.2)
Parity	Primiparous	8(47.1)
	Multiparous	9(52.9)

Data analysis resulted in three broad themes: experiences of mistreatment, perceived drivers, and consequences of mistreatment in health facilities. Codes under experiences of mistreatment were organised into categories aligned with the WHO’s typology of mistreatment [33], such as verbal and physical abuse, stigma, and discrimination–classified as interpersonal abuse within the Socioecological Framework [21]. Other categories, including privacy violations, poor rapport, neglect and abandonment, and system constraints were grouped under mistreatment in the care process, aligning with the broader Quality of Care Framework [22]. The drivers and the consequences of mistreatment were similarly developed, with drivers analysed across individual, interpersonal, health facility, health system, and societal levels. Overall findings are presented within an integrated conceptual framework combining the Socioecological and the WHO’s Quality of Care Frameworks, as illustrated in Fig 3.

### Narratives of mistreatment experienced

To understand women’s experiences in health facilities, women were asked about their initial expectations from health facilities and what they found during their maternity care. Participants disclosed that all they required from both health facilities and healthcare providers was to obtain quality services that consider their psychological well-being aligning with health facilities’ mission—“*initial promises*”, as they expressed. For instance, while describing the services that women or the community as a whole require from health institutions and professionals, the following interview participant said:

“*…What I mean is, irrespective of a person’s circumstances, it would be nice if they greeted people with a friendly demeanour. It would be ideal if health care providers understood what patients are going through and treated them with the same warmth and empathy, in support of their mental wellness.”* [IDI-WP-09]

The participants also emphasised the importance of receiving quality care, care that maintained professional, solid ethics. The experiences during antenatal care and private clinics were described as ‘*respectful’* and ‘*caring*.*’* While describing the behaviours of health care providers, a 20-year-old primiparous woman referred to antenatal care service providers as *“courteous”* and mentioned them as an example of polite, well-mannered providers:

*“…Um, I think … if they assist people in a good way instead of treating people disrespectfully, the women who come there are like their mothers and sisters, and they should be treated with kindness… yea, like the place where we received antenatal care, they were very helpful. We are very grateful to them; they were very courteous.”* [IDI-WP-08]

One woman who switched from a public hospital to private care due to her past negative experiences described her experience at the private clinic as excellent. She expressed it as follows:

*“I opted for private care for birth because of the bad history I had previously. In the private facility, they have taken excellent care of me, provided medication, and ensured a smooth birth. After giving birth, they continued to care for my baby.”* [IDI-WP-06]

#### Categories of mistreatment experienced by women

As participants shared their expectations and positive experiences with healthcare providers and facilities, the topic of mistreatment emerged spontaneously. Participants described the negative experiences they had encountered, witnessed, or heard about, either secondary to interpersonal abuse or as a lack of quality of care during the care process, as presented accordingly in the following sections.

#### Interpersonal abuses

*Physical abuse*. Women participants raised concerns about physical abuse, describing instances where they had observed healthcare providers resorted to hitting women during childbirth due to perceived non-compliance. Participants also highlighted encountering painful procedures as a result of receiving pain-relieving analgesics or anaesthesia, as well as forceful shoving. While sharing her negative experience, a 20-year-old woman described the behaviour of a healthcare provider who assisted in her birth, stating, *“She hit my thighs”* and “*tossed me back and forth*.*”*.

*“When she assisted with my birth, she hit my thighs with her hands and tossed me back and forth on the bed. She had the practice students take turns watching me. She told me that my baby was distressed and cut my body [episiotomy]. But she got up and moved away from me; she didn’t seem to be trying to save my distressed baby. … she stepped away from me and said, ‘Finish screaming first!’.”* [IDI-WP-08]

Furthermore, instances of forceful pushing, hitting, or pinching with forceps during the second stage of labour were reported, either from personal experience or witnessing another woman being hit. One participant stated, *“They hit you with scissors”* and *“pinch you*,*”* and mentioned that she had personally experienced being struck by a healthcare provider [IDI-WP-06].

Even when it might not be intended to cause harm, forceful pushing and aggressively placing a woman’s legs apart have been reported in situations where a woman did not comply with the orders given by health care providers. When asked if she had experienced anger or any physical aggression from healthcare providers, one participant described the situation as follows:

*“If you scream or try to lift your legs from where they have positioned them, they will forcefully push your legs back down. This was clearly obvious.”* [IDI-WP-02]

Lack of provision of local anaesthetic during perineal incision for episiotomy, as well as the omission of other forms of pain-relieving agents during instrumental birth and uterine wiping, were reported to cause excruciating pain and discomfort for the women undergoing these procedures. Women who experienced such incidents suggested providers justified carrying out these painful procedures as a means to prevent the complications and fear of being responsible for poor outcomes, as witnessed in the underneath quote:

*“The experience of restitching after the birth was very painful…She tried to clean me up, going in and out with her hands. This is what I still find troubling. The pain was intense when she reached into my sensitive womb.’”* [IDI-WP-03]

In addition to their personal experiences, some women also witnessed other women being physically abused. One woman described observing another woman being hit because she was tired and was unable to find the energy to push during childbirth:

*“I witnessed them hitting another labouring woman at my side, but not me. … I saw her being beaten because she could not progress.”* [IDI-WP-02]

*Verbal abuse*. Women detailed experiencing various forms of verbal abuse, including scolding, threatening, mockery, blame, disavowals, and other insulting remarks from health care providers and other supportive staff in the facility. They described enduring such verbal abuses throughout their stay in the healthcare facility, beginning from their initial visit for antenatal care for some women and often during labour at the time of birth. For instance, one woman reported being scolded, “*You are not even showing*!” when she first visited the hospital and then mentioned having to leave the hospital in a disappointed mood.

Participants frequently also mentioned being yelled at in derogatory terms, which they described as making them feel unwelcome. One mother described the unsympathetic and insensitive behaviour of a healthcare provider during labour, stating:

*“My labour pain gets severe, and my husband came and held me up… ‘What! Why are you hanging on to her? Let her go. Is she the only one experiencing labour pain!’ said one provider to my husband as he supported me while I was in labour pain. Because it was my first time, I did not know how to handle the pain. After that, they became four [providers], and they said, ‘Her labour is not that severe; she does not require a bed,’ as they stood beside me, adding, ‘Is she superior to the rest? Why did the doctor assign her a bed?’ "* [IDI-WP-01]

Another primiparous mother shared her experience of being scolded mockingly throughout her birthing process, including targeted personal comments related to her appearance. She also talked about how her childbirth experience lacked informative support. Instead of receiving gentle and professional guidance, the healthcare providers were aggressive, and their communication style felt like yelling, which only added to the woman’s distress during labour, making the situation *“quite worrisome*.*”*

*“Right from the beginning, you approach the situation[labour] with a sense of anxiety. When the person working there shows anger, it becomes quite worrisome. Uhm…they express their scolds loudly, telling you not to shout and to push quietly. Throughout my birth, I heard her saying, "The baby is small, that’s all she was struggling for."* [IDI-WP-02]

Health facilities, particularly hospitals, are sometimes overcrowded, and there may be insufficient resources to accommodate them. In these situations, women have reported experiencing being yelled at, feeling ignored, and receiving negative verbal responses when requesting post-natal beds. One woman recalled staying on the birthing couch throughout the night after giving birth and only being able to get up in the morning. When they asked for a bed, the healthcare providers responded saying:

*‘There are no beds; Should I give birth to it for you?’.”* [IDI-WP-10]

Another form of verbal abuse reported by women includes being threatened and blamed for any negative outcomes that have occurred or were feared to be possible. These incidents were observed when women failed to cooperate with healthcare providers, indicating a lack of responsibility for any potential complications and depicting the provider’s attitude as ‘passing the buck’. For instance, one woman mentioned that she was told that if anything happened to her, they would not be liable. So, she needed to endure the pain and follow the provider’s instructions:

*“. When I pleaded, ‘Please, it is painful,” she responded, ‘Then sign a release so I won’t be held responsible if you face any issues.’… I just watched. I wanted it to be over.”* [IDI-WP-03]

Another woman also described such experience as follows:

*“There are times when they say, ‘Spread your legs,’ and they purposely scold you even when you ask the right thing …and I take these all as serious problems.”* [IDI-WP-04]

A mother who had lost her previous baby a few hours after birth described the situation, recalling her experience. She recounted how healthcare providers blamed her for her baby’s feeding issues and discharged her without properly assessing the newborn’s condition. While describing the situation she faced during the loss of her previous baby, she recounted it as follows:

*“… when we informed them about the baby’s difficulty in sucking, one of the healthcare providers there came and inserted her finger into the baby’s mouth and said, ‘The baby is healthy, but it is you who refused to breastfeed.’ Afterwards, they asked us to leave… they discharged us even though we informed them that the baby was not breastfeeding. They said, ‘Unless you have refused to give her, the baby is healthy and can latch.’ She never had a proper latch and never breastfed.”* [IDI-WP-06]

*Stigma and discrimination*. Several women reported feeling discriminated against or receiving a lower level of attention compared to others. One woman reported perceiving discrimination when an attendant provided unequal care, favouring a non-labouring woman over those in distressing labour. She expressed frustration and concern over this unequal treatment, suggesting the reason for the preferential care might be related to personal connection or the role played by woman’s notability. This differential treatment left the participant feeling disappointed and frustrated:

*“I wanted to raise the issue of treating people unequally… I saw a nurse sitting next to a woman … cared well. I wished for such care. I know that person, maybe she is a famous person. While another mother was screaming in labour, the nurse was sitting and chatting with that woman without enquiring what was happening with the other woman screaming‥”* [IDI-WP-02]

In relation to overcrowding in the facility, women reported that healthcare providers controlled and restricted birth companions in the labour and birthing ward but that this rule was applied inconsistently. Partiality in enforcing rules, possibly due to familiarity between clients and staff or other factors, left some women feeling neglected, helpless, unsupported, and confused during childbirth. One woman felt isolated and believed she was treated differently when it came to having a birth companion and described that the health care provider did not consistently enforce the same rule for everyone; they allow birth companions for some while they send other birth companions away.

*“While sending out my companion, she did not do this for all… She would go and help the other woman in labour, saying, ‘Cheer up, be strong.’ I don’t know if they knew each other or not. She even called someone for her, her companions, to help her, and they would reach out to her, offering encouragement. She was very helpful for her, but for me…. puu, nope.”* [IDI-WP-08]

#### Mistreatment in the process of care

The mistreatment experienced by women in this category is not solely attributed to the individual behaviours of healthcare providers. Instead, these instances have emerged within the care process, stemming from compounded issues involving healthcare providers and the facility—in the lack of quality care perspective. These issues include violations of privacy, non-consented care, lack of confidentiality, neglect, abandonment, and poor communication. Delays in receiving midwifery care, lack of attention and breaches of professional standards of care led to some women enduring life-threatening situations, as depicted below:

“*They discharged me while I was still bleeding, before my condition had stabilised, and before I had changed my clothes. I gave birth at 8 o’clock, and I was discharged at 12 o’clock. They mentioned they would provide the necessary items like vaccination for the baby immediately after birth. However, later, as they saw me still lying there, they asked, "What are you doing here for so long?" So, I just left at that point.”* [IDI-WP-05]

*Violation of privacy*, *confidentiality*, *and preferences*. Another set of issues related to the structural arrangements of the labour and birthing unit, as well as the attitude of the health care providers, pertained to the failure to meet professional standards of care. This included the violation of privacy, confidentiality and the inability of women to participate in the decisions being made about their care. One woman, despite describing the service she received as ’good,’ highlighted the lack of privacy and consented care during examinations:

“*At the time they examined me, there were no curtains or screens. The way they accommodate you is good, but there was no privacy. When they examine my cervix, they remain silent, and no one asks you for your consent or preferences.”* [IDI-WP-04]

This lack of consent was not an isolated incident and was often accompanied by practices that violated the privacy of women during labour or childbirth. Associated with the uncomfortable setup of the health care facility, such as the absence of curtains or screens and private rooms, there were reports of multiple people being present in a single small labour attendance unit. When a woman was asked if healthcare providers obtained her consent or provided an explanation before performing an episiotomy, she described that it was not the case and mentioned hearing the sound of cutting her body without explanation:

*"…when I was giving birth … I recall being instructed to ’push … push.’ I believe I was able to push the baby out, but while I was following their directions, they made an incision in my body, I just heard the ’snip’ sound while they cut my body without saying anything to me. …They did not ask me anything of the sort. Around seven or eight apprenticeship students were standing around me, observing. She did not say anything to me–no explanation, no mention of doing stitches, and no pain relief medication.”* [IDI-WP-03]

Besides the violation of privacy and confidentiality, women also reported unnecessary repeated examinations along with poor communication. One woman, while explaining that she had never experienced such mistreatment in her previous births, stated that several students repeatedly performed vaginal examinations on her without offering any explanations, all in front of the people present in the room.

*"They repeatedly examined me without saying anything. Even many of the students who were practising there have also seen me right in front of the people, in front of everyone who came to visit other birthing women. There were many people present, and many women were lying; in front of everybody, they performed examination [vaginal examination]."* [IDI-WP-05]

*Neglect and abandonment*. Feeling neglected and abandoned was identified as additional forms of mistreatment described by women during their care, attributed either to individual healthcare providers’ attitudes or facility conditions. Some women emphasised that certain providers would occasionally ignore them, potentially worsening the women’s conditions, which they reported as *’feeling left alone’*, *’ignored’*, *’neglected’* and *’abandoned’*, not only when they were alone but also when healthcare providers were present, expressing a sense of inattentive observation or using the woman’s unborn baby as a tool to gain compliance. One woman shared her experience:

*“… She turned to me and said, ‘Your baby is dying, be strong.’ But she moved away and started sitting. She told me that my fetus was distressed and cut my body [episiotomy]. But she got up and moved away from me, she did not seem to be trying to save my distressed baby.”* [IDI-WP-08]

When the provider who initially admitted the mother left her alone, other providers often claimed that it was not their responsibility. This abandonment or lack of attention during labour led to women giving birth by themselves in the same place where they were labouring:

*"I gave birth on the same bed I was lying on when I first arrived, and they did not even notice when I gave birth. I gave birth to my baby on my own pyjamas… It was not until the people in the room shouted, ’Please, she’s giving birth here,’ that the healthcare providers rushed in. When they arrived, I had already given birth right there. My child had water [amniotic fluid] in his nose. He even still struggles to breathe."* [IDI-WP-05]

The participants also reported instances where labouring mothers were intentionally abandoned, ignored, or neglected by the providers. One woman recounted an experience where she felt abandoned by a group of providers due to hierarchical issues among them. She mentioned that one provider prevented others from examining her during labour, insisting that she wanted to be examined only by a specialist doctor, contrary to her actual desire. She recalled it as:

*“When another doctor came to examine me, one of them said, ‘Leave her alone; there is no one other than the specialist doctor who checks her status.’ She did this at least to two, and then they left me…while they came and assessed other labouring women repeatedly, they just ignored me.”* [IDI-WP-01]

*Exposed to unfair fees*. Maternal and child health services in public facilities in Ethiopia are generally exempt from charges. However, some women have reported being unexpectedly subjected to outsourced fees. One woman complained that she was asked to purchase medications but did not receive them.

*“Initially, they sent my husband to buy medication. After he made the purchase, they took it away and never returned it. We bought medication for 500 Ethiopian Birr and left without receiving it. They repeatedly asked him to bring medicine.”* [IDI-WP-05]

### Perceived drivers of mistreatment

As participating women discussed their experiences during service utilisation, they also shared their perceptions of the drivers of these mistreatments. These perceived drivers within this theme were categorised based on their nature, ranging from individual to system and community-level drivers based on the Socioecological Framework and the WHO quality of care frameworks as presented in the following sections.

#### Ontogenic: Individual-level factors

Issues at the individual level that the women thought to be influential in the occurrence of mistreatment include *exhaustion*, *burnout*, *negligence*, *negative attitudes*, and *fear of poor outcomes*, among others. One participant, reflecting on her own experiences during childbirth, mentioned that a "*lack of empathy*" and "*attitude*" might have contributed to those behaviours rather than increased workloads. She also recognised that there were respectful providers among them when asked about the aspects contributing to these negative behaviours.

*"I am not sure why they would behave like that. They might mention being busy, …umm…but you see only a few patients in the hospital when they themselves are being crowded. If it were really crowded, you would understand …I think it is a lack of empathy…Yes, their attitude is somewhat lacking. However, there are some who genuinely care for people and provide dedicated service. There are individuals who have strong desire among them."* [IDI-WP-05]

On top of the impacted empathy and lacking attitude, exhaustion leading to despair was also perceived as one of the underlying reasons for mistreatment by other women. Participants brought up the issue of exhaustion, which may affect the confidence and satisfaction that providers have in their service provision and their professional outlook when dealing with individuals of various personalities:

*"I think it is because they work there every day, and it makes them exhausted. They, the workers, seem desperate. I do not think they find satisfaction in performing their skilled tasks. Uhm … it looks like they lose their way and end up there in despair due to the intense experiences they go through. I wonder if these experiences with women could affect them and lead to improper behaviours. Maybe these lead them to be careless."* [IDI-WP-03]

Negligence arising from carelessness was also mentioned as a reason behind the behaviour of some providers and leading to mistreatment. Such behaviours overall are described as a loss of responsibility and accountability.

*"I would say the problem lies in the lack of accountability. It is not just the health care professionals but everyone working in the system who needs to take responsibility. I think they have forgotten their responsibilities. …. Even those who are passionate about their job and understand the excitement of a new life coming into the world seem to have forgotten this."* [IDI-WP-06]

#### Microsystem: Interpersonal

Interpersonal issues, whether between women and healthcare providers or among professionals themselves, were identified as contributing to negative experiences for women in healthcare facilities. One of these interpersonal issues contributing to mistreatment was poor communication during interactions. For instance, a woman who felt impatiently treated by a midwife [the women mostly call/see midwives as nurses] during a labour examination reported that misunderstandings between her attendants and healthcare providers resulted in her being ignored by the care team. She recounted the midwife’s nervousness as:

*“It was my mother who accompanied me to the labour ward initially; after we arrived there, I was sitting, and my mother was standing. Then they asked us…’what was your case?’ my mother did not hear what they asked and replied, ‘uhh, what did you say?’ to the nurse who asked us because she didn’t hear what the nurse asked as she was facing toward me, and then the nurse said ‘what does it mean to say ’uhh’, just leave the room and stay outside’, while getting nervous [laughter…].”* [IDI-WP-01]

#### Mesosystem: Health facility-related issues

Various issues related to health facility conditions, ranging from health facility types, lack of conducive environment, facility-specific policies, crowdedness, delay and resource-related issues, were perceived by participants to act as a conduit for the occurrence of mistreatment. Some women raised concerns related to health facility policy, including a lack of an adequate number of providers during holidays, poor regulation and control from the leaders, and lack of a redress system, which all result in mistreatment. When recounting the way providers hand over cases, one woman said she was left alone because the one who was caring for her left without reporting to others:

“*I got tired of asking for help. Those who were supposed to assist me just disappeared, abandoning me, let alone helping me without my consent. Others would say, ‘She’s not mine; she belongs to Sister ’X’, did she go off?’ by calling her name. They did come after many requests for help… I believe they swapped shifts, maybe she left during that time.”* [IDI-WP-02]

A woman who believed that addressing women’s complaints could enhance the health facility’s shortcomings noticed a lack of initiatives aimed at assessing these weaknesses and striving for improvement. She observed a deficiency in feedback mechanisms from both health centres and the hospitals’ leadership, which could have been used to address facility weaknesses in providing care.

*“The organisation should address community complaints. I think that covers it. Unfortunately, in many places, there seems to be a lack of follow-up, and it appears that the leadership has forgotten its responsibilities. I haven’t come across that. I’ve never seen any inquiries about whether we were receiving proper service or not, neither in the health centre nor in the hospital.”* [IDI-WP-06]

Lack of readiness of health centres in terms of resources was also cited to enhance occurrences of mistreatment by leading women to undermine services provided in health centres and leading them to opt for hospitals, resulting in the crowdedness of the hospital.

*“There are some differences between the conditions at the health centre and the hospital…It is difficult to say there is follow-up at the health centre; the hospital is more equipped. It was only iron that you get from the health centre.”* [IDI-WP-04]

#### Exosystem: Health system

Several issues stemming from different levels of the health system influence the services offered to women by healthcare providers and facilities. These concerns are connected to the foundational aspects of quality care within the healthcare system, encompassing both organisational and physical elements that shape the care process. Overcrowding in health facilities, conflict arising because of hierarchy among providers, fear of blame, disabled functionality of the referral system, culture of blame, and lack of accountability among providers were highlighted to drive mistreatment.

Power dynamics among providers marked by poor coordination, a lack of teamwork and respect often leave women feeling isolated and abandoned. These issues reflect deep-rooted problems in interdisciplinary collaboration, shaped by professional training norms and hierarchical positions, such as those between doctors and midwives:

*“… the providers became four, and they said, ‘Her labour is not that much, and it does not necessitate a bed.’ As they stood beside me, they added, ‘Is she superior to the rest? Why did the doctor assign her a bed?’”* [IDI-WP-01]

The fear of being held responsible for any negative outcomes that women and newborn babies might experience is found to influence how healthcare providers behave. Some women reported being threatened by professionals, who warned they would not be held responsible if anything went wrong with them or their babies. This behaviour reflects broader systemic issues, where the focus is placed on avoiding adverse outcomes, potentially at the expense of the quality of care provided. This issue is highlighted in a quote from a woman who underwent a painful procedure:

“*… There’s a saying from providers, "If something happens to the child, it’s not on me.*" [IDI-WP-04]

#### Macrosystem: Society level

This category presents how societal factors, such as gender norms, beliefs, and community attitudes, influence how health facilities and healthcare providers deliver services. Structural gender inequality in society leads to the tolerance of violence against women. Consequently, mistreatment of women becomes normalised within health facilities, stemming from the belief that ’health care providers have the right to chastise women’ during childbirth to ensure compliance with the birthing process. These circumstances render women in a passive role, disempowered from speaking out against abusive behaviours during service reception. Such normalisation and disempowerment of women could be seen when one woman recounted an incident where another woman was bitten and expressed the futility of lodging complaints by stating, ’no one hears you’:

*“…may be the hospitals are different, but in another hospital, I saw two midwives beating a woman who was bleeding while a tube was in her body, I saw it with my own eyes… when I witnessed those two midwives beating that woman, I wanted to retaliate physically if I could … if I were in that situation, I do not think I would stay quiet…What else can you do? No one hears you.”* [IDI-WP-05]

Insecurity within the community due to ongoing unrest was another contextual issue found to have a substantial impact on the services tailored for women. This included restricted access and the reluctance of service providers, ultimately disrupting the referral system linking lower-tier facilities with higher ones. Reflecting on the challenges amplifying the constraints of rural health centres and health posts, a woman highlighted the scarcity of ambulances and instances where health facilities remained inaccessible despite undertaking lengthy journeys to reach semi-urban district facilities.

*“In our village? … [yes …], the problem is the one I mentioned to you now. If a pregnant woman goes into labour and if one calls for an ambulance, they’ll tell you, ‘The ambulance was burned.’ Even for the labouring woman herself, if they find a private car, they pay and take her to the facilities.”* [IDI-WP-07]

### Consequences of mistreatment of women

In response to inquiries about the repercussions of mistreatment within health facilities, participants expressed a spectrum of outcomes. These included psychological impacts, discontinuation of care, and a heightened fear of childbirth, all influencing their decisions regarding ongoing service utilisations.

*“…Some opt to deliver at home, saying, ‘It is better to stay at home than going hospital and get incised and sutured.’ Why do you go to the health facility? Isn’t it for better care, both for yourself and the baby? … If there is no better care at facilities; those with money go for private birth, while those without may choose to give birth at home, and I know someone who refused to go and gave birth at home.”* [IDI-WP-06]

Another woman highlighted the impact of mistreatment on both the fear surrounding childbirth and the reputation of the health facility within the community, expressing her determination not to revisit the facility.

*“We talk about it at home, ’maybe they were busy.’ Even if they were busy, it shouldn’t be like this. I have made up my mind not to go there again. It has completely turned me away from seeking their services, as to me.”* [IDI-WP-02]

Some mentioned that mistreatment prompted them to choose private clinics, affecting the reputation of public health facilities and affecting. For those women who could not afford a private clinic or hospital, their alternative option to birthing in a public hospital was to give birth at home alone. One mother vividly described how having experienced mistreatment at one health facility, she contemplated avoiding giving birth at a health facility, stating:

*“…on the first day, I went home because of the scolds. I mean, I made up my mind and decided home birth. My family objected and called for an ambulance. I think it might be the same for other people.”* [IDI-WP-09]

In addition to driving women away from public health facilities, it was also reported to be related to the unfortunate death of newborns. A woman who experienced mistreatment in her previous birth mentioned that the outcome of her baby could have been different if the providers had responded to their requests:

*“…I lost my child due to mishandling issues like this. If they had provided proper care during birth and had assisted us when we said the baby wasn’t breastfeeding, the outcome might have been different.”* [IDI-WP-06]

## Discussion

This study explored the narratives of women’s experiences, perceived drivers and consequences of mistreatment of women during maternity care in line with the Socioecological and WHO Quality of Care Frameworks, following the initial development of themes from the women’s in-depth interviews in East Wollega Zone, Western Ethiopia. The findings show the persistence of the mistreatment of women despite the attention given to eliminating it both globally and nationally [4, 14].

The narratives of mistreatment reported by women, whether from their own experiences or observations of other women, include interpersonal abuse and mistreatment during the care process. Among these forms of mistreatment, the first three—physical abuse, verbal abuse or stigma and discrimination can be considered as intentional interpersonal abuse occurring between women and healthcare professionals and should be viewed as a subset of the violence against women in the community [21]. The consideration that mistreatment of women during maternity care aligns with violence against women in society is supported by previous work by Jewkes et al. [11]. Physical abuse, as reported by women in this study, includes acts of hitting, slapping, forceful pushing, and denial of pain-relieving agents. These are intentional actions, even if the motives of healthcare providers may stem from preventing bad outcomes rather than inflicting harm [34]. Similarly, verbal abuse, which encompasses negative comments, insults, mockery, and derogatory words, was also reported along with stigma and discrimination, which often arise from providers’ prejudices. These prejudices were perceived by women to originate from various factors such as favouritism toward individuals of higher social status (e.g., education and economic status), familiarity between the woman and healthcare providers, and partiality in implementing health facility policies (e.g., those related to birth companions). Such factors were identified as reasons predisposing women to discriminatory actions, consistent with previous studies [17, 35, 36]. These experiences of women have also been reported previously in earlier qualitative studies [36–40], highlighting the sustained suffering of women during pregnancy and birth from interpersonal abuse.

The remaining forms of mistreatment align with health system failures and issues encountered during the processes of caregiving, which are closely related to the failure of overall quality of care [22]. Women reported being ignored or abandoned when requesting help, even when expressing concerns about complications. Some of these forms of mistreatment stemmed from deliberate malevolence, such as the collective abandonment of women by groups of professionals after labelling them as non-cooperative or non-respectful behaviour towards an individual midwife. Likewise, examining a woman in front of others without visual and auditory privacy, including confidentiality violation and failing to obtain informed consent before procedures, were also reported. Structural issues, such as the absence of curtains and the use of shared rooms, may have contributed to such mistreatment in the care process, as also mentioned in previous studies [33, 41]. In most Ethiopian health facilities, it is common for many students from various disciplines to attend to a single birthing mother in non-emergency situations, exacerbating overcrowding and repeatedly violating women’s rights to dignified care and autonomy. These practices reinforce a lack of respect for privacy and informed consent, which should be recognised as breaches of women’s autonomy, impacted professionalism and a challenge to the quality of care.

While there should be no justifications for mistreatment, multiple underlying factors were perceived as contributing to its occurrence. These driving factors manifest from various levels, including personal history and characteristics, interpersonal issues, concerns within healthcare facilities, broader health system factors, and societal influences, mirroring the pattern seen in general violence against women in the community [21]. Personal history or the individual characteristics of healthcare providers, such as exhaustion, demotivation, and burnout, can lead to negligence and reduced empathy, directly ending with mistreatment. Such behaviours from healthcare providers may stem from high workloads and limited staffing [38]. This implies that personal issues, when compounded with chronic health facility and system-related issues, worsen the mistreatment of women in health facilities. Women’s perception of poor-quality care at health centres, accompanied by the lack of resources and limited staff, leads them to bypass these centres and opt for hospitals, resulting in overcrowding and increasing the risk of mistreatment or neglect in emergencies [28]. As expressed by study participants, health centres often remain empty due to these perceptions, with women fearing inadequate care and the unavailability of essential resources. Consequently, women prefer hospitals, exacerbating overcrowding and contributing to mistreatment.

In addition to personal and health facility-level factors, mistreatment can result from the interaction of drivers at macro-level factors—originating from societal norms and health system cultures [11]. In Ethiopia, a significant proportion of women experience various forms of violence, including early unconsented marriage, multiple forms of domestic and intimate partner violence, and poor societal perception of women’s education and work rights [26]. These societal factors influence the healthcare providers’ perceptions of women, compounded by health system issues such as inadequate recognition and unfair treatment from the leaders towards providers. When healthcare providers themselves feel disrespected by health system administrators and facility leaders, power dynamics strain relationships [42, 43]. This can lead providers at lower levels to exert control over women through mistreatment actions, including verbal or physical abuse, as also witnessed in a previous study [44].

During pregnancy and childbirth, women are vulnerable and often unable to defend and protect themselves from mistreatment. This mistreatment does not leave them unaffected; it can result in serious consequences for both the mother and the child [3]. In this study, women reported various forms of consequences of mistreatment, including fear of childbirth in health facilities and switching to giving birth at home while knowing the consequences of unattended birth at home in the hands of unskilled relatives traditionally, failures of the reputations they have for health facilities and healthcare providers, and even neonatal loss. The findings also suggest that mistreatment led to psychological distress, with women reporting the effects of interpersonal abuse as well as feelings of being ignored and abandoned. Such instances of mistreatment signify a lack of psychological support during labour and birth and have been linked to postpartum depression in previous studies [45, 46]. Neglect or abandonment during labour and birth can result in devastating outcomes, including stillbirth, neonatal death, and maternal death if unanticipated obstetric emergencies, such as fetal distress or severe bleeding encounter. These devastating consequences of mistreatment highlight the urgency of prioritising this issue and necessitate policy intervention to ensure respectful and dignified care for all, regardless of circumstances.

The interviews conducted with women have provided valuable insights from diverse perspectives. However, despite efforts to engage participants from various community groups within the Zone, our initial targets were not fully met due to civil unrest in certain areas during data collection. We endeavoured to include rural participants through mobile phone interviews to address this challenge. However, conducting interviews via telephone limited our ability to grasp the nuanced nonverbal cues that often enrich communication and understanding. The benefits of conducting face-to-face interviews would have included an observation of nonverbal cues throughout the interviews, which could have led to further discussion in understanding the depth of negative consequences of mistreatment of women.

## Conclusions

This qualitative study presents women’s first-hand experiences of mistreatment in health facilities, where three main themes were explored: the experiences of mistreatment, the drivers of mistreatment, and the consequences of mistreatment in East Wollega Zone, western Ethiopia among women who received maternity care from health facilities. These experiences lead to significant negative consequences and implications on service delivery. The findings highlight the need to understand the complex factors contributing to mistreatment, which go beyond individual healthcare providers’ behaviours to macro–level health systems issues and an acceptance of societal violence against women. This emphasises the importance of applying a systems-thinking approach to address the abuse and suffering women experience when receiving maternity care in health facilities.
